# Supplementary material for: Lymphotoxin α fine-tunes T cell clonal deletion by regulating thymic entry of antigen-presenting cells
Source: Nat Commun. 2018 Mar 28;9:1262. doi: 10.1038/s41467-018-03619-9 (PMC5872006; doi:10.1038/s41467-018-03619-9)
Supplement: Supplementary file 1 — Supplementary Information(PDF 3325 kb) [file 41467_2018_3619_MOESM1_ESM.pdf]

Supplementary information:

Lymphotoxin α fine-tunes T cell clonal deletion by regulating thymic entry of antigen-presenting cells

Lopes *et al.*

Supplementary Figure 1

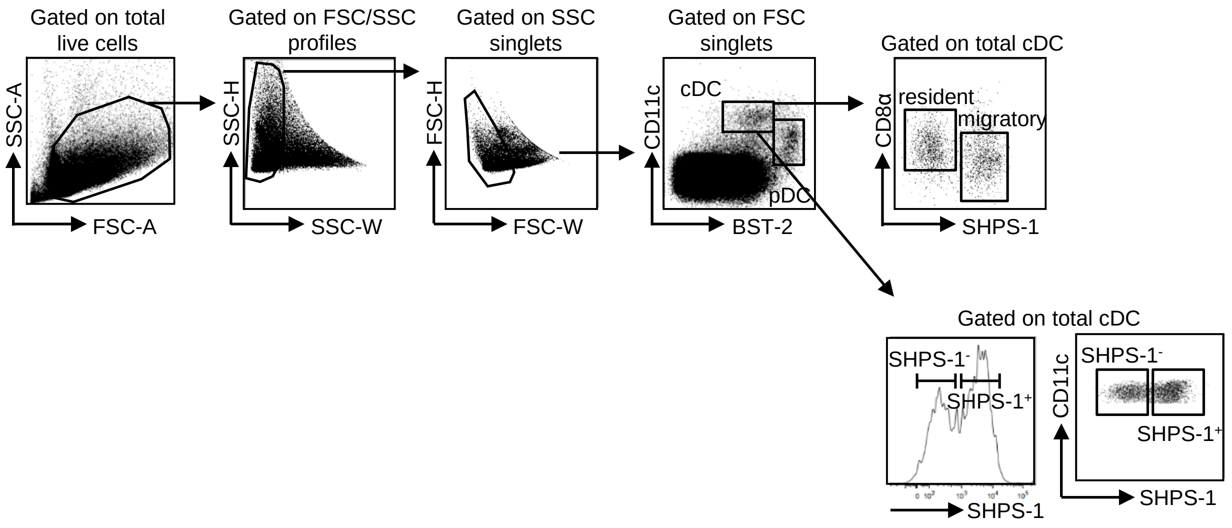

Supplementary Figure 1. Gating strategy used to analyse thymic DC subsets.

Total thymic cDCs and pDCs were identified as $CD11c^{hi}BST\text{-}2^{lo}$ and $CD11c^{int}BST\text{-}2^{hi}$ cells, respectively. Resident and migratory cDCs were further identified among the total cDC population as $CD8\alpha^{hi}SHPS\text{-}1^{-}$ and $CD8\alpha^{lo}SHPS\text{-}1^{+}$ cells, respectively. Alternatively, resident and migratory cDCs were identified as $SHPS\text{-}1^{-}$ and $SHPS\text{-}1^{+}$ cells among the total cDC population. The same gating strategy was used to analyse DC subsets in Fig. 1a, b, d, g; Fig. 2b, c, g; Fig. 4b, e; Fig. 7b, d, e. Supplementary Fig. 2b, c, e; Supplementary Fig. 3; Supplementary Fig. 4a, c; Supplementary Fig. 5a, b; Supplementary Fig. 6c; Supplementary Fig 7b; Supplementary Fig. 8a, b, d, e; Supplementary Fig. 9; Supplementary Fig. 10; Supplementary Fig. 11a, b; Supplementary Fig. 12b, Supplementary Fig. 14a.

Supplementary Figure 2

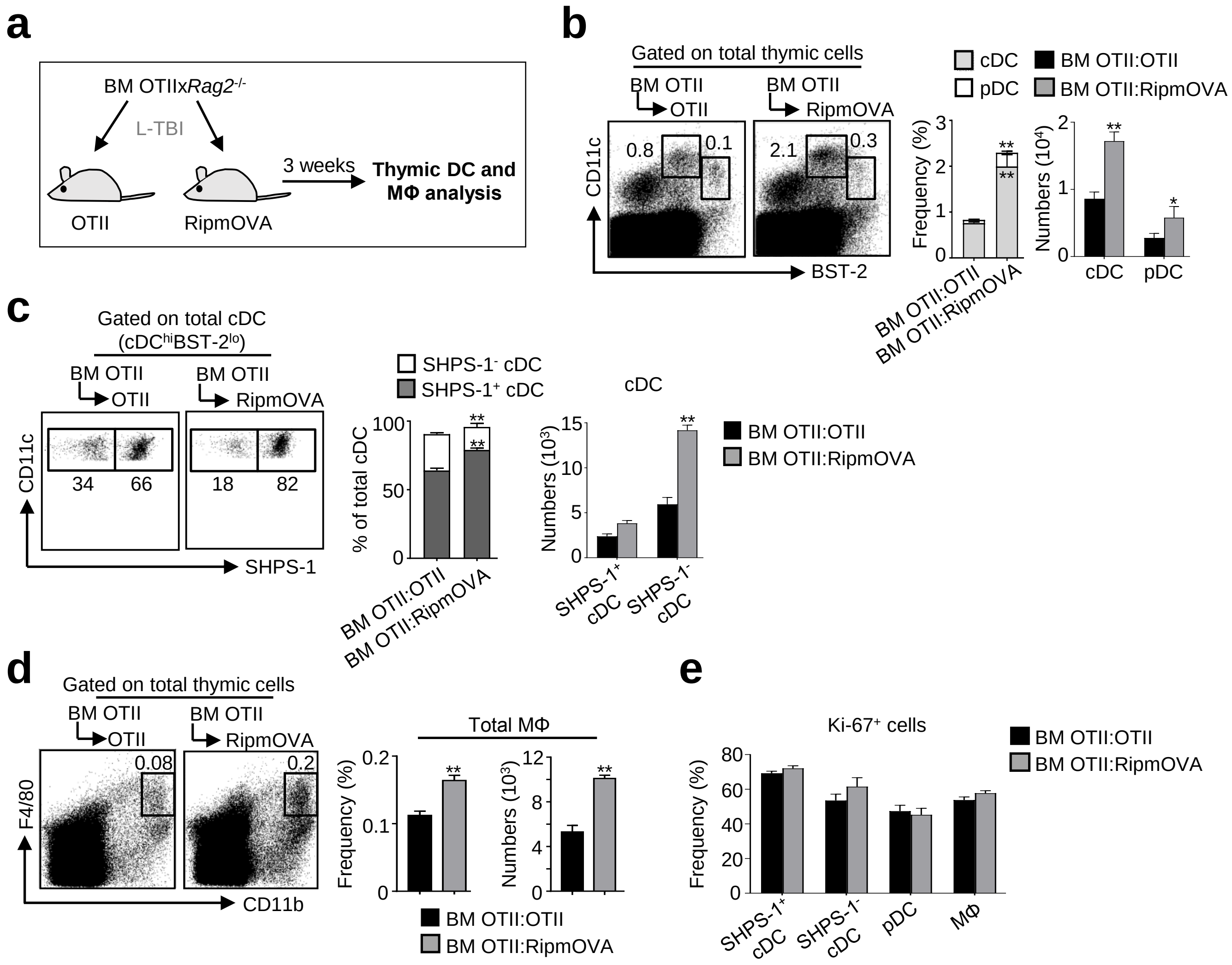

Supplementary Figure 2. Crosstalk between CD4⁺ thymocytes and OVA-expressing stromal cells increases numbers of peripheral DCs and macrophages into the thymus.

(a) Experimental setup: Lethally irradiated OTII-*Rag2*$^{-/-}$ and RipmOVA-*Rag*2$^{-/-}$ recipient mice were transplanted with BM cells from OTII-*Rag2*$^{-/-}$ mice (OTII:OTII and OTII:RipmOVA chimeras, respectively). Three weeks after BM transplantation, DCs and macrophages were analysed in the thymus. L-TBI; lethal total body irradiation. **(b-d)** Flow cytometry profiles, frequencies and numbers of cDCs (CD11c^{hi}BST-2^{lo}), pDCs (CD11c^{int}BST-2^{hi}) **(b),** SHPS-1^{-} resident cDCs, SHPS-1^{+} migratory cDCs **(c)** and macrophages (F4/80^{+}CD11b^{+}) **(d)** in the thymus from OTII:OTII and OTII:RipmOVA chimeras. **(e)** The histogram shows the frequencies of proliferating Ki-67^{+} thymic DC subsets and macrophages. **(b-e)** Data are representative of two independent experiments (n=3 mice per group and per experiment). **(a, d, e)** MΦ; Macrophage. Error bars show mean ± SEM, **(b-d)** *p<0.05, **p<0.01 using two-tailed Mann-Whitney test.

Supplementary Figure 3

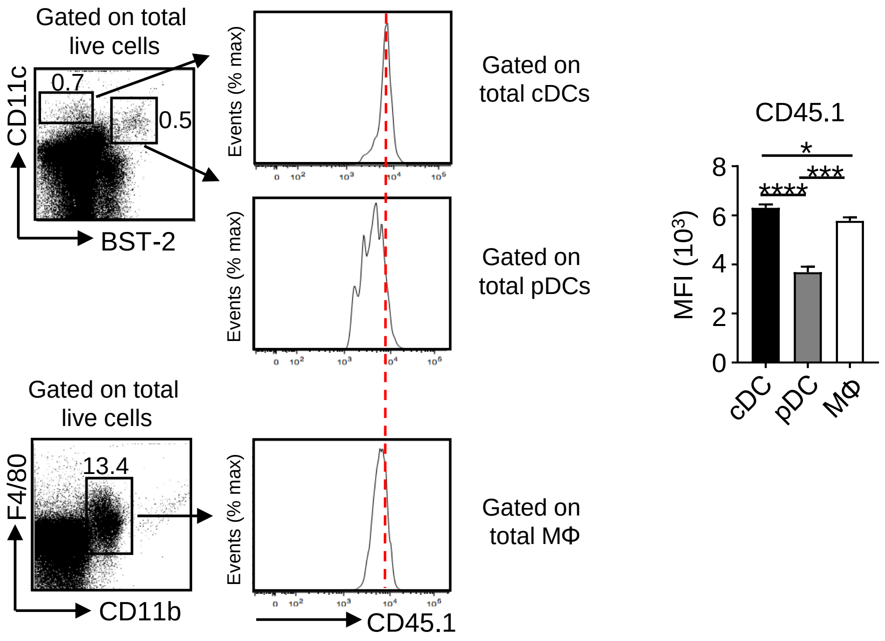

Supplementary Figure 3. WT blood circulating pDCs express lower levels of the CD45.1 congenic marker than cDCs and macrophages.

CD45.1 expression level was analysed by flow cytometry on blood cDCs (CD11c^{hi}BST-2^{-}), pDCs (CD11c^{int}BST-2^{hi}) and macrophages (F4/80^{+}CD11b^{+}) from CD45.1 WT congenic mice. The red doted line allows comparing CD45.1 expression level in the different cell types analysed. The histogram shows the MFI values of CD45.1 staining normalized to the FMO value of each population analysed. MFI: Mean fluorescence intensity. Data are representative of two independent experiments (n=3 mice per group and per experiment). MΦ; Macrophage. Error bars show mean ± SEM, *$p<0.05$, ***$p<0.001$, ****$p<0.0001$ using unpaired Student's t-test.

Supplementary Figure 4

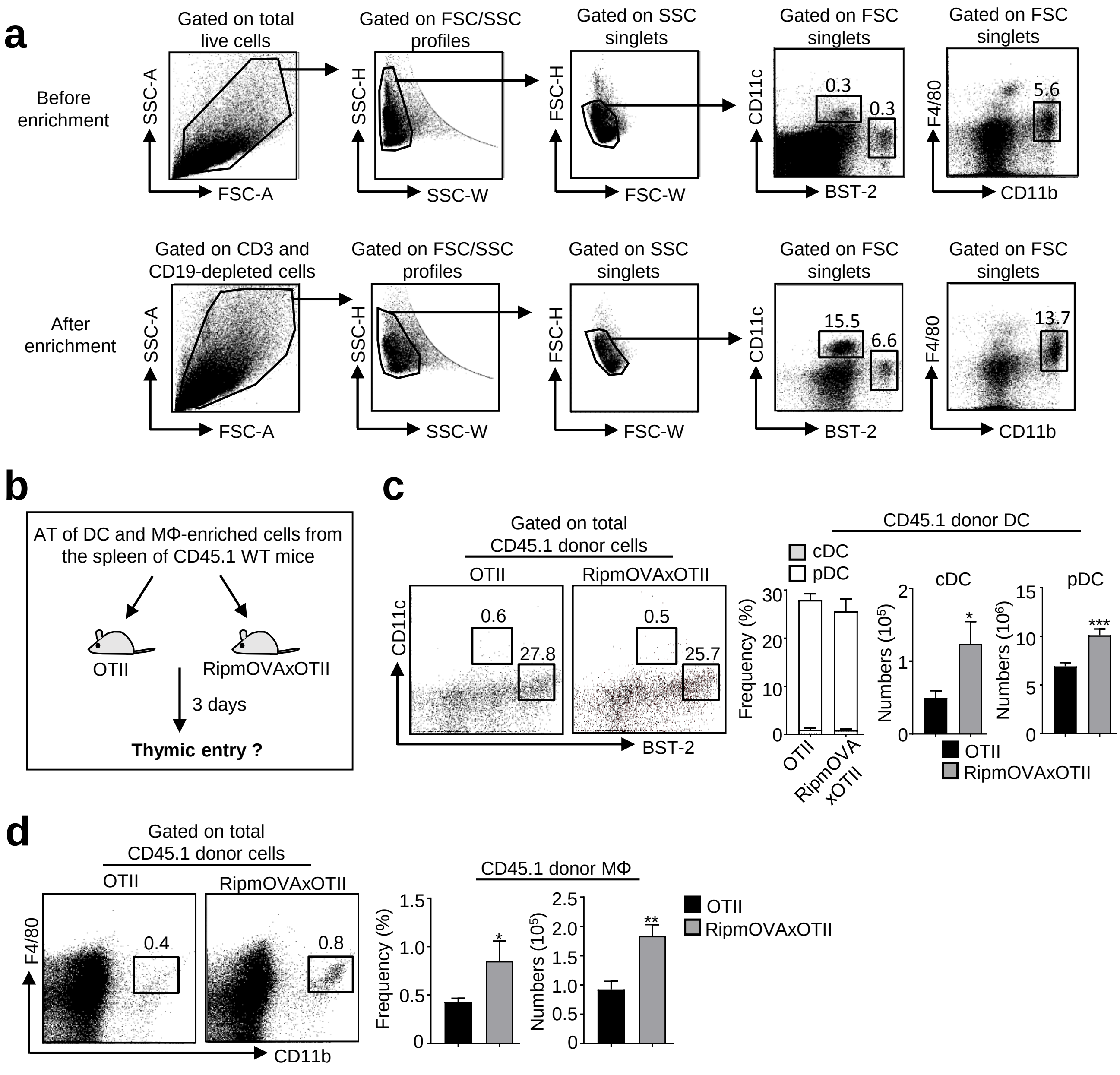

Supplementary Figure 4. Thymic entry of donor peripheral DCs and macrophages is enhanced upon mTEC-CD4^{+} thymocyte crosstalk.

(a) The gating strategy used to verify the enrichment of cDCs (CD11c^{hi}BST-2^{lo}) pDCs (CD11c^{int}BST-2^{hi}) and macrophages (F4/80^{+}CD11b^{+}) from the spleen of CD45.1 congenic mice by depletion of CD3^{+} and CD19^{+} cells is shown. The same strategy was used to enrich splenic DCs and macrophages from CD45.1 congenic mice in Supplementary Fig. 7. **(b)** Experimental setup: DC and macrophage-enriched cells from the spleen of CD45.1 WT congenic mice were adoptively transferred into nonirradiated CD45.2 OTII-*Rag2*$^{-/-}$ and RipmOVAxOTII-*Rag2*$^{-/-}$ recipient mice. Three days after *i.v.* adoptive transfer, the thymic entry of DCs and macrophages of CD45.1 donor origin was analysed. **(c,d)** Flow cytometry profiles, frequencies and numbers of cDCs (CD11c^{hi}BST-2^{lo}), pDCs (CD11c^{int}BST-2^{hi}) **(c)** and macrophages (F4/80^{+}CD11b^{+}) **(d)** of CD45.1 donor origin. **(b-d)** Data are representative of two independent experiments (n=3-4 mice per group and per experiment). **(b, d)** MΦ; Macrophage. Error bars show mean ± SEM, *p<0.05, **p<0.01, ***p<0.001 using unpaired Student's t-test.

Supplementary Figure 5

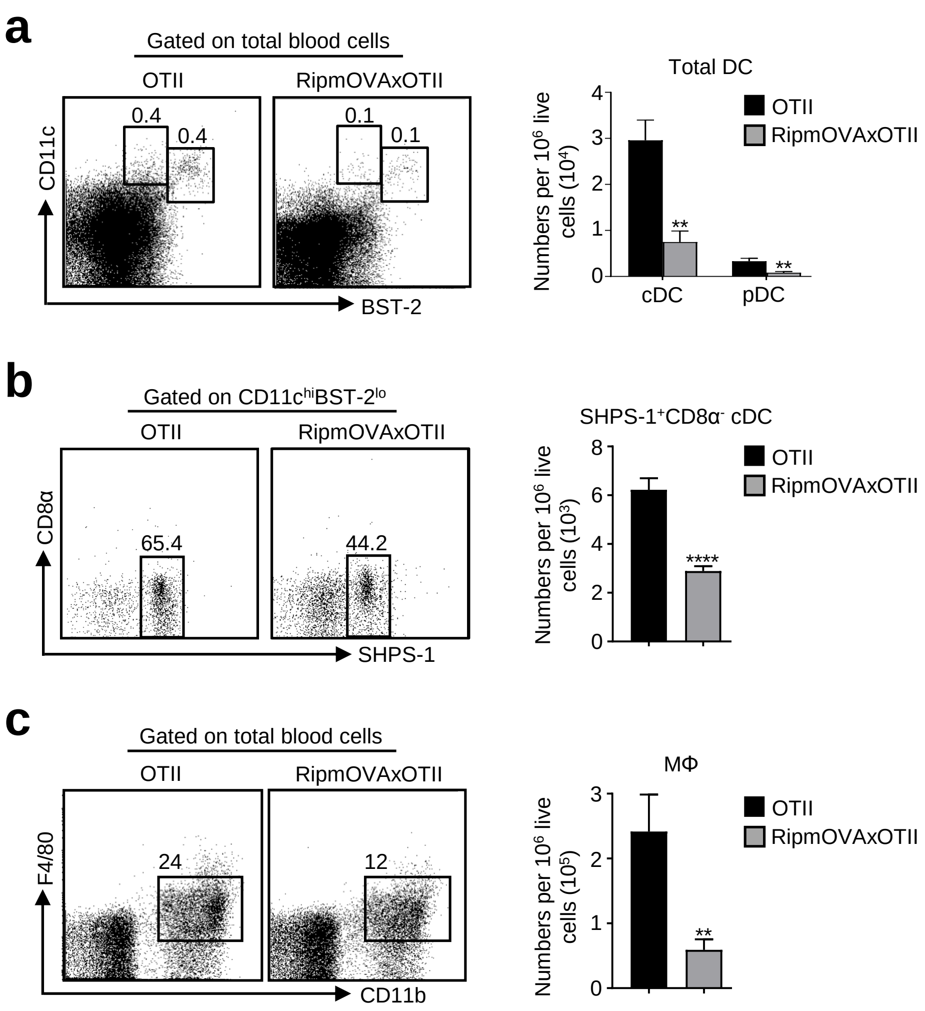

Supplementary Figure 5. Reduced numbers of DCs and macrophages in the blood from RipmOVAxOTII-*Rag2*$^{-/-}$ mice compared to OTII-*Rag2*$^{-/-}$ mice.

Flow cytometry profiles and numbers of cDCs (CD11c^{hi}BST-2^{lo}), pDCs (CD11c^{int}BST-2^{hi}) **(a)**, migratory cDCs (CD11c^{hi}BST-2^{lo}SHPS-1^{+}CD8α^{-}) **(b)** and macrophages (F4/80^{+}CD11b^{+}) **(c)** in the blood from OTII-*Rag2*$^{-/-}$ and RipmOVAxOTII-*Rag2*$^{-/-}$ mice. Data are representative of three independent experiments (n=3 mice per group and per experiment). **(c)** MΦ; Macrophage. Error bars show mean ± SEM, **p<0.01, ****p<0.0001 using unpaired Student's t-test.

Supplementary Figure 6

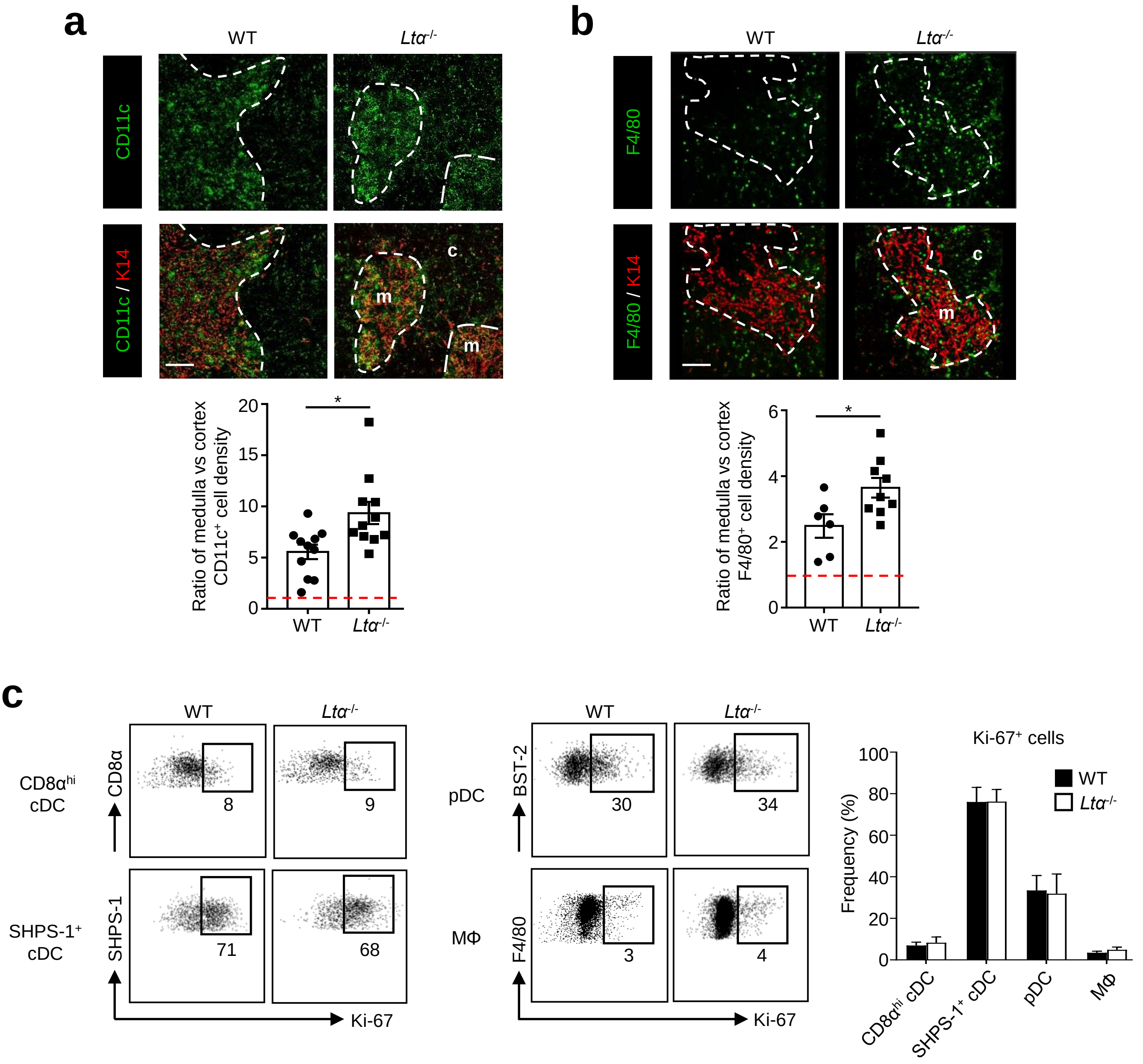

Supplementary Figure 6. DCs and macrophages are highly enriched in thymic medullary regions from *Ltα*$^{-/-}$ mice but show a normal cellular proliferation.

(a, b) Thymic sections from WT and *Ltα*$^{-/-}$ mice were stained with antibodies against either CD11c (green) and K14 (red) **(a)** or F4/80 (green) and K14 (red) **(b)**. m and c denote the medulla and cortex, respectively. Graphs show quantifications of the ratio of medullary versus cortical density of CD11c^{+} and F4/80^{+} cells. Each dot represents an individual confocal image derived from two to three mice for each genotype. The red dotted line denotes a ratio of 1, corresponding to the same density in the cortex and medulla. Scale bar, 100 µm. **(c)** Flow cytometry profiles and frequencies of Ki-67^{+} proliferating CD8α^{hi} cDCs (CD11c^{hi}CD8α^{hi}SHPS-1^{-}), SHPS-1^{+} cDCs (CD11c^{hi}CD8α^{lo}SHPS-1^{+}), pDCs (CD11c^{int}BST-2^{hi}) and macrophages (F4/80^{+}CD11b^{+}) in the thymus from WT and *Ltα*$^{-/-}$ mice. Data are representative of four independent experiments (n=3 mice per group and per experiment). **(c)** MΦ; Macrophage. Error bars show mean ± SEM, *p<0.05 using unpaired Student's *t*-test.

Supplementary Figure 7

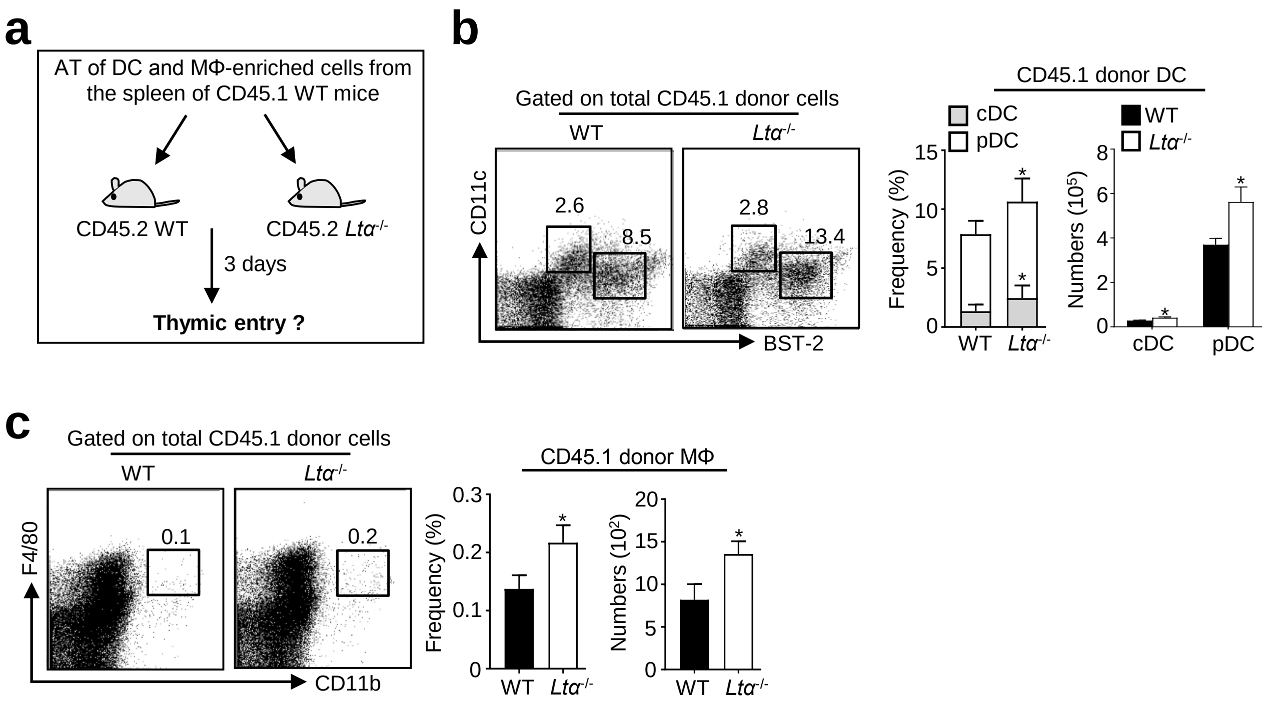

Supplementary Figure 7. LTα negatively regulates the thymus homing of peripheral DCs and macrophages.

(a) Experimental setup: DC and macrophage-enriched cells purified from the spleen of CD45.1 WT congenic mice were adoptively transferred into nonirradiated CD45.2 WT and *Ltα*$^{-/-}$ recipients. Three days after *i.v.* adoptive transfer, the thymic entry of DCs and macrophages of CD45.1 donor origin was analysed. **(b, c)** Flow cytometry profiles, frequencies and numbers of cDCs (CD11c^{hi}BST-2^{lo}), pDCs (CD11c^{int}BST-2^{hi}) **(b)** and macrophages (F4/80^{+}CD11b^{+}) **(c)** of CD45.1 donor origin. Data are representative of three independent experiments (n=3-4 mice per group and per experiment). **(a-c)** MΦ; Macrophage. Error bars show mean ± SEM, *p<0.05 using unpaired Student's t-test.

Supplementary Figure 8

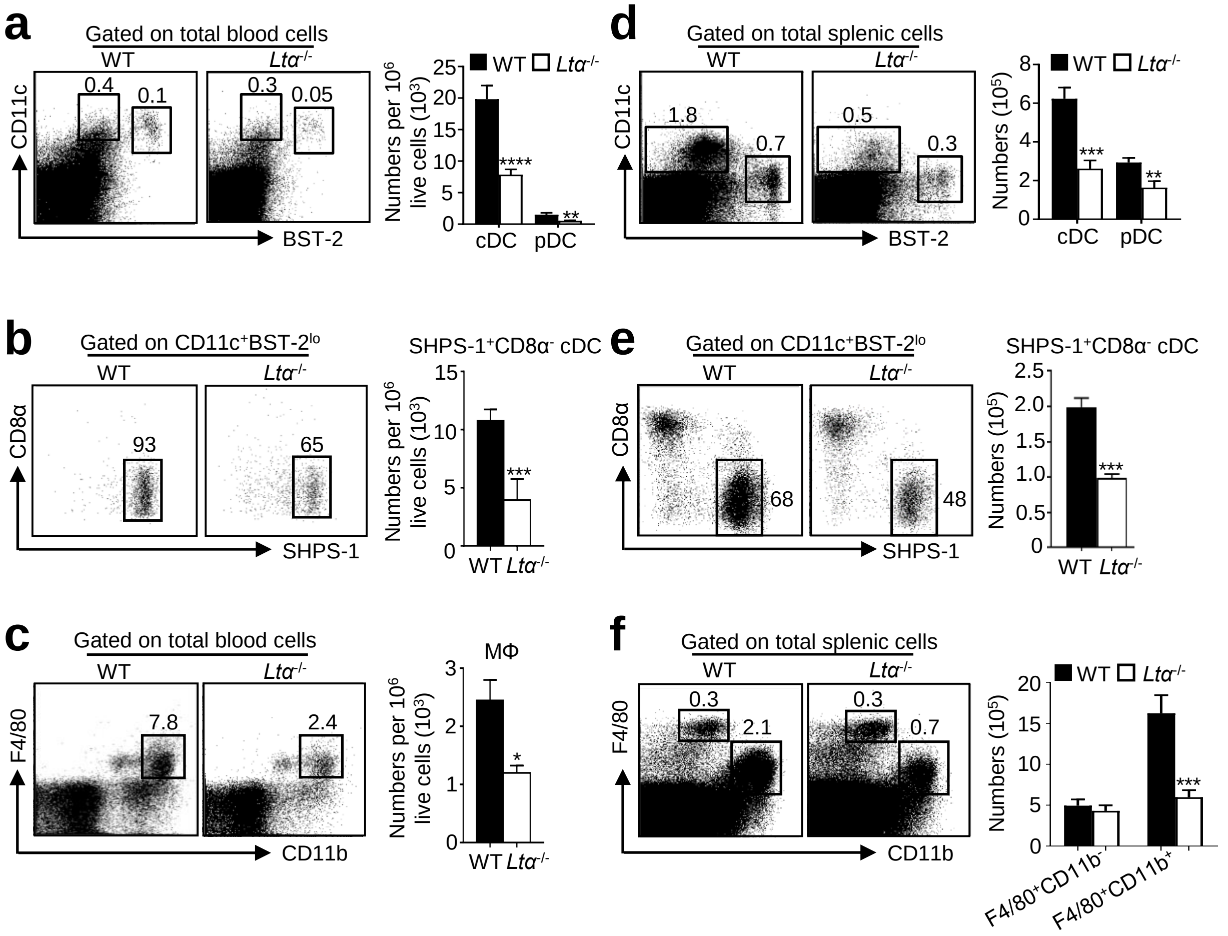

Supplementary Figure 8. Reduced numbers of DCs and macrophages in the blood and spleen of *Ltα*$^{-/-}$ mice.

Flow cytometry profiles and numbers of cDCs (CD11c^{hi}BST-2^{lo}) and pDCs (CD11c^{int}BST-2^{hi}) **(a, d)**, migratory cDCs (CD11c^{hi}BST-2^{lo}SHPS-1^{+}CD8α^{lo}) **(b, e)** and macrophages (F4/80^{+}CD11b^{+}) **(c, f)** in the blood **(a-c)** and spleen **(d-f)** of WT and *Ltα*$^{-/-}$ mice. Data are representative of three independent experiments (n=3 mice per group and per experiment). **(c)** MΦ; Macrophage. Error bars show mean ± SEM, *p<0.05, **p<0.01, ***p<0.001, ****p<0.0001 using unpaired Student's t-test.

Supplementary Figure 9

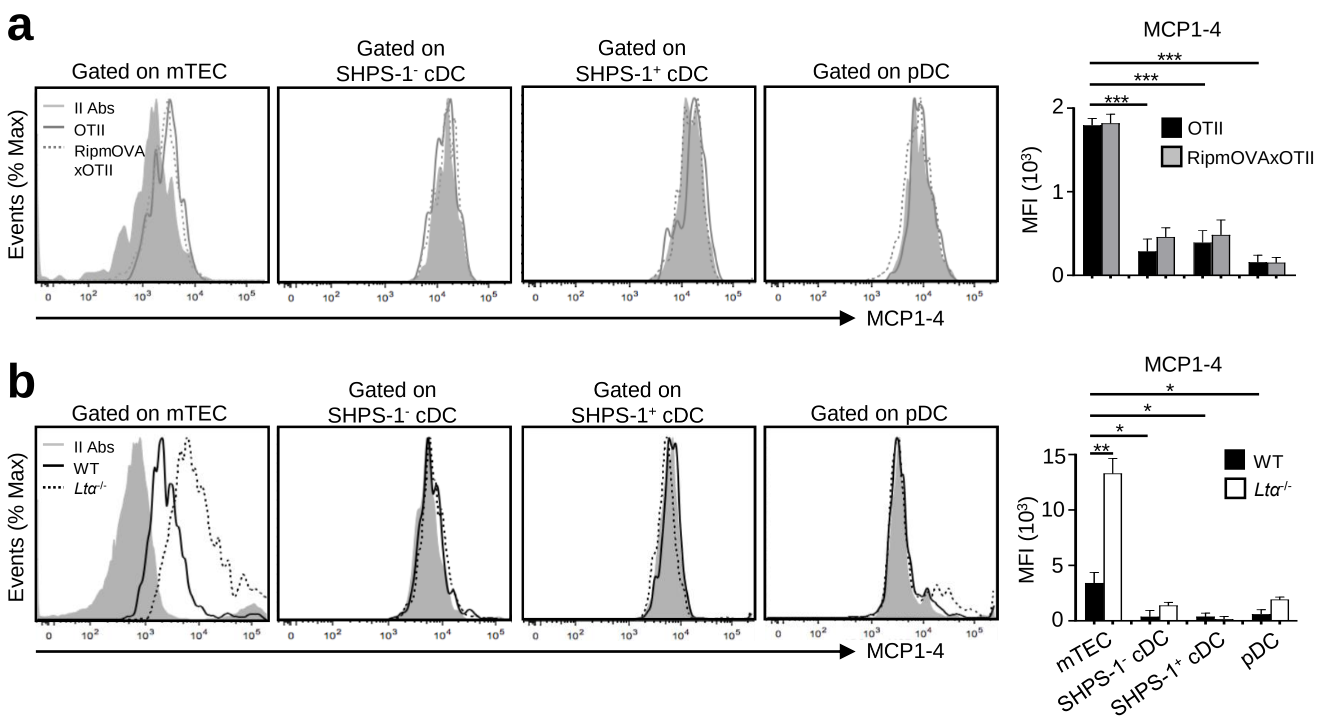

Supplementary Figure 9. MCP1-4 proteins are expressed in mTECs but not in thymic DC subsets.

MCP1-4 protein expression was analyzed by flow cytometry in purified mTECs (Ep-CAM^{+}UEA-1^{+}BP-1^{lo}), resident cDCs (CD11c^{hi}BST-2^{lo}CD8α^{hi}SHPS-1^{-}), migratory cDCs (CD11c^{hi}BST-2^{lo}CD8α^{lo}SHPS-1^{+}) and pDCs (CD11c^{int}BST-2^{hi}) in the thymus from either OTII-*Rag2*$^{-/-}$ and RipmOVAxOTII-*Rag2*$^{-/-}$ mice **(a)** or WT and *Ltα*$^{-/-}$ mice **(b)**. Histograms show the MFI values of MCP1-4 staining normalized to secondary antibody values of each population analysed. MFI: Mean fluorescence intensity; II Abs: Secondary Antibodies. Data are representative of two independent experiments (n=3-4 mice per group and per experiment). Error bars show mean ± SEM, *p<0.05, **p<0.01, ***p<0.001 using unpaired Student's t-test.

Supplementary Figure 10

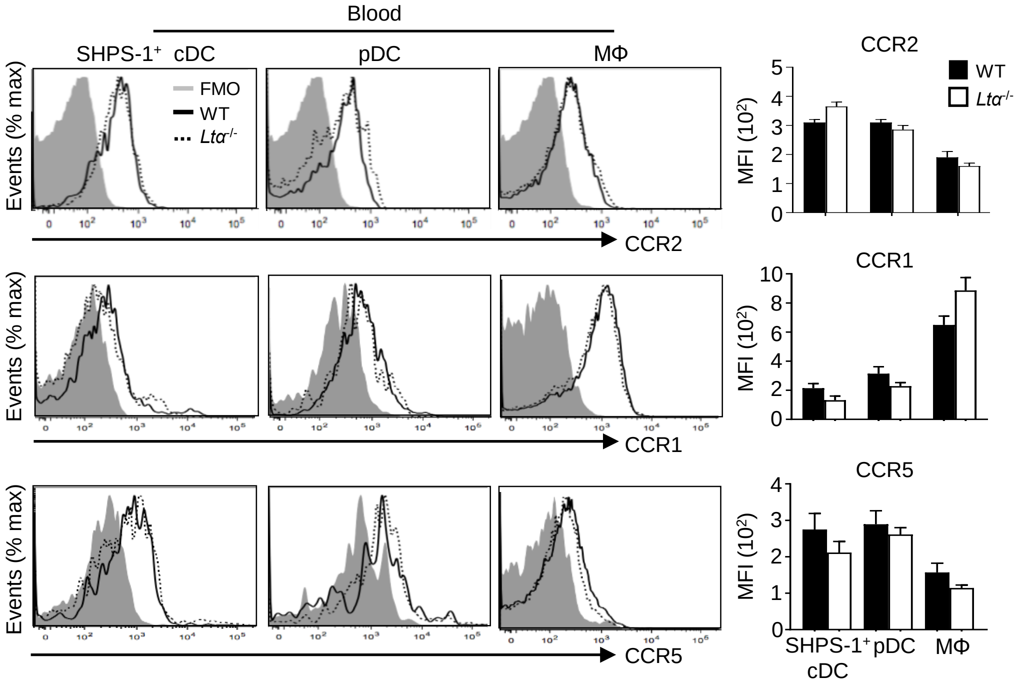

Supplementary Figure 10. CCR2, CCR1 and CCR5 expression on circulating DCs and macrophages in WT and *Ltα*$^{-/-}$ mice.

Cell surface expression of CCR2, CCR1 and CCR5 on migratory cDCs (CD11c^{hi}BST-2^{lo}SHPS-1^{+}CD8α^{lo}), pDCs (CD11c^{int}BST-2^{hi}) and macrophages (F4/80^{+}CD11b^{+}) was analysed by flow cytometry in the blood of WT and *Ltα*$^{-/-}$ mice. Histograms show the MFI values of CCR2, CCR1 and CCR5 staining normalized to the FMO value of each population analysed. MFI: Mean fluorescence intensity, FMO: Fluorescence Minus One. Data are representative of two independent experiments (n=5 mice per group and per experiment). MΦ; Macrophage. Error bars show mean ± SEM.

Supplementary Figure 11

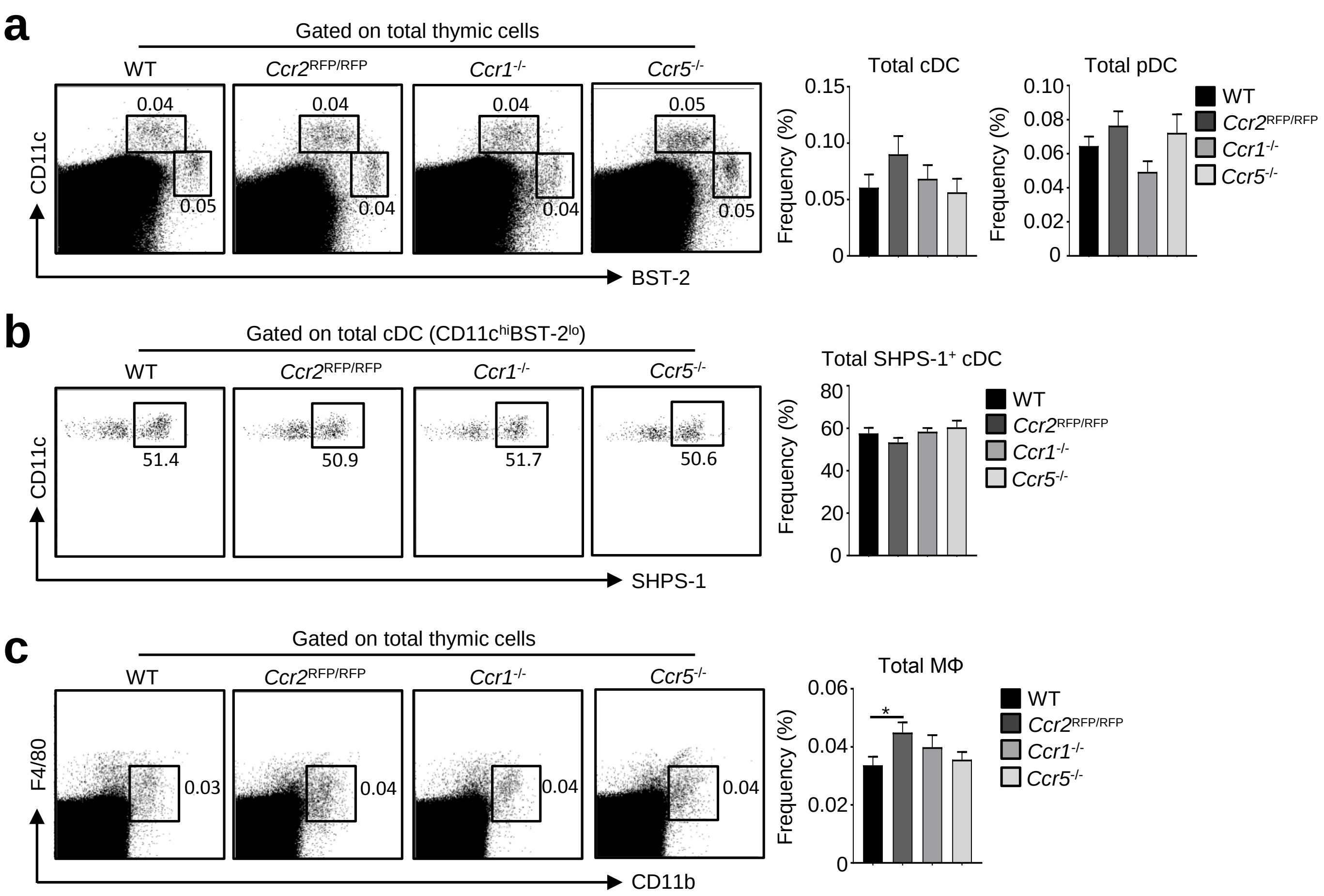

Supplementary Figure 11. Gating strategy used to analyse cDCs, pDCs and macrophages in the thymus of mixed BM chimeras for WT, *Ccr2*$^{RFP/RFP}$, *Ccr1*$^{-/-}$ and *Ccr5*$^{-/-}$ donor groups.

Mixed BM chimeras were performed as described in Fig. 4a. **(a-c)** Flow cytometry profiles and frequencies of total cDCs (CD11c^{hi}BST-2^{lo}), pDCs (CD11c^{int}BST-2^{hi}) **(a)**, migratory cDCs (CD11c^{hi}BST-2^{lo}SHPS-1^{+}) **(b)** and macrophages (F4/80^{+}CD11b^{+}) **(c)** in the thymus of WT, *Ccr2*$^{RFP/RFP}$, *Ccr1*$^{-/-}$ and *Ccr5*$^{-/-}$ donor groups. Data are representative of two independent experiments (n=4-5 mice per group and per experiment). **(c)** MΦ; Macrophage. Error bars show mean ± SEM; *p<0.05 using unpaired Student's t-test.

Supplementary Figure 12

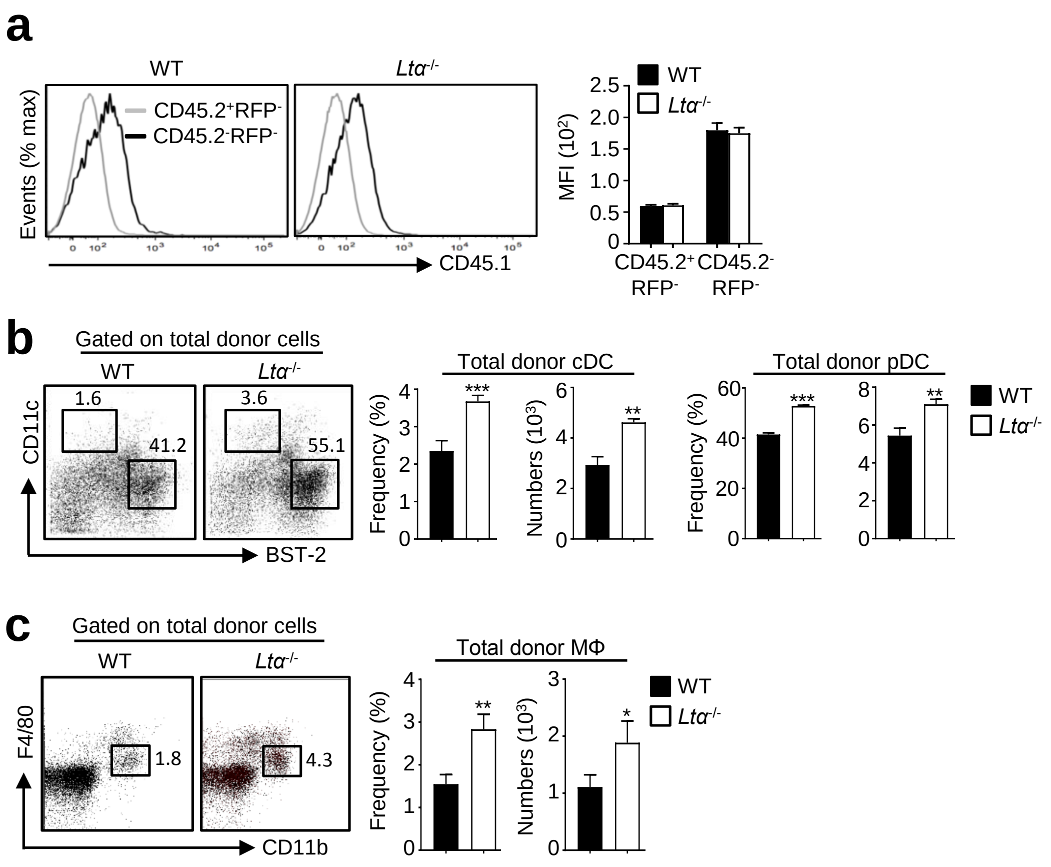

Supplementary Figure 12. Increased frequencies and numbers of peripheral DCs and macrophages derived from total donor cells in the thymus of WT and *Ltα*$^{-/-}$ mice.

(a) CD45.1 expression level in CD45.2^{+}RFP^{-} endogenous cells and CD45.2^{-}RFP^{-} donor cells in WT and *Ltα*$^{-/-}$ recipient mice. MFI: Mean fluorescence intensity. **(b,c)** Flow cytometry profiles, frequencies and numbers of cDCs (CD11c^{hi}BST-2^{lo}), pDCs (CD11c^{int}BST-2^{hi}) **(b)** and macrophages (F4/80^{+}CD11b^{+}) **(c)** of total donor origin (CD45.2^{-}RFP^{-} and CD45.2^{+}RFP^{+} cells) in the thymus of WT and *Ltα*$^{-/-}$ mice. **(a-c)** Data are representative of two independent experiments (n=4 mice per group and per experiment). **(c)** MΦ; Macrophage. Error bars show mean ± SEM, *p<0.05, **p<0.01, ***p<0.001 using unpaired Student's t-test.

Supplementary Figure 13

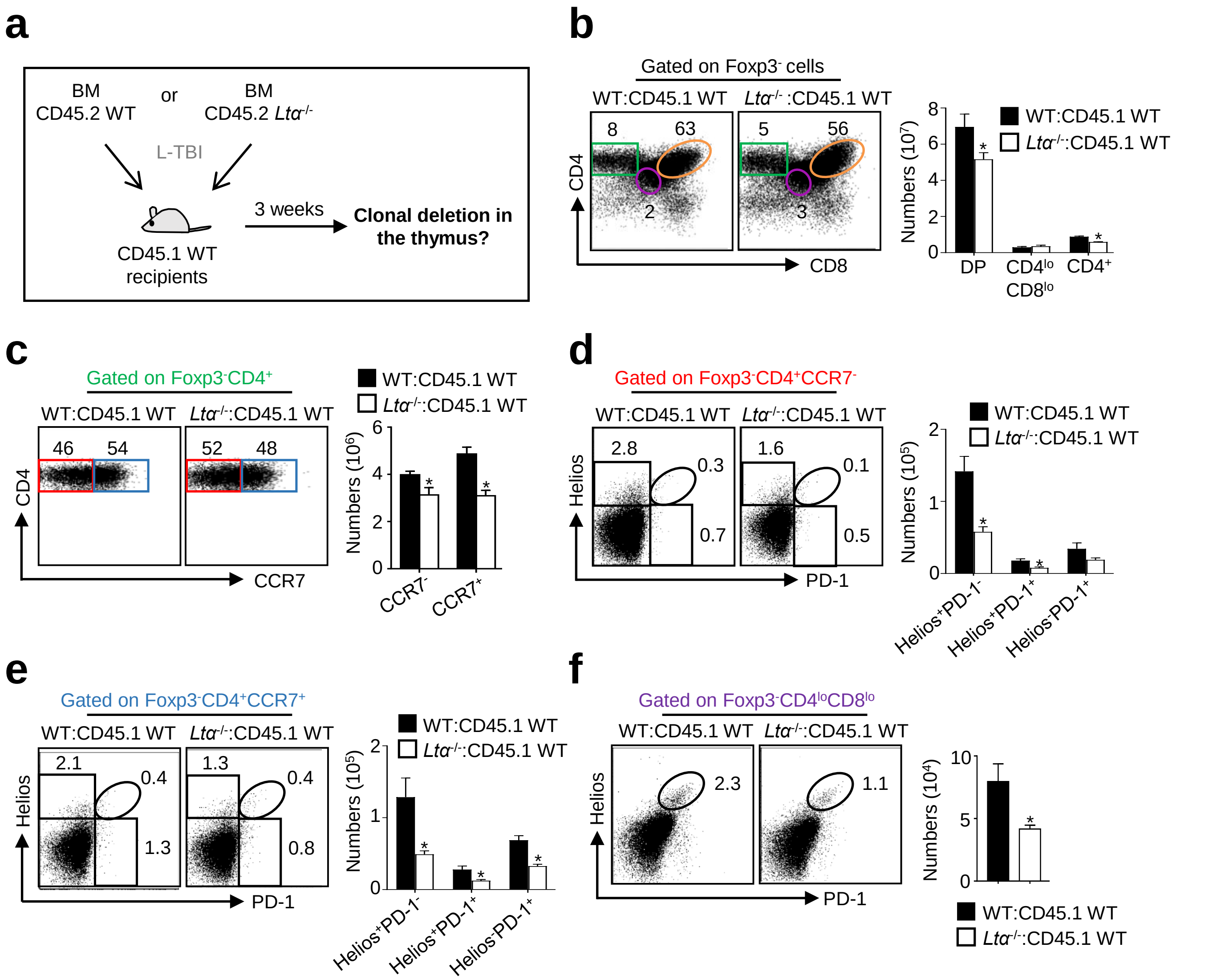

Supplementary Figure 13. Enhanced clonal deletion in the thymus from *Ltα*$^{-/-}$:CD45.1 WT chimeras.

(a) Experimental setup: Lethally irradiated CD45.1 WT recipient mice were transplanted with BM cells from CD45.2 WT or *Ltα*$^{-/-}$ mice (WT:CD45.1 WT and *Ltα*$^{-/-}$:CD45.1 WT chimeras, respectively). Three weeks after BM transplantation, clonal deletion was analysed in the thymus. L-TBI; lethal total body irradiation. **(b,c)** Flow cytometry profiles and numbers of DP (CD4$^+$CD8$^+$), CD4^{lo}CD8^{lo} and CD4$^+$ (CD4$^+$CD8$^-$) cells analysed in Foxp3$^-$ cells **(b)** and CCR7$^-$ and CCR7$^+$ cells analysed in Foxp3$^-$CD4$^+$ SP cells **(c)** from the thymus of WT:CD45.1 WT and *Ltα*$^{-/-}$:CD45.1 WT chimeras. **d-f** Flow cytometry profiles and numbers of Helios$^+$PD-1$^-$, Helios$^+$PD-1$^+$ and Helios$^-$PD-1$^+$ in Foxp3$^-$CD4$^+$CCR7$^-$ **(d)**, Foxp3$^-$CD4$^+$CCR7$^+$ **(e)** and Foxp3$^-$CD4^{lo}CD8^{lo} cells **(f)** from the thymus of WT:CD45.1 WT and *Ltα*$^{-/-}$:CD45.1 WT chimeras. Data are representative of two independent experiments (n=4 mice per group and per experiment). Error bars show mean ± SEM, *$p<0.05$ using two-tailed Mann-Whitney test.

Supplementary Figure 14

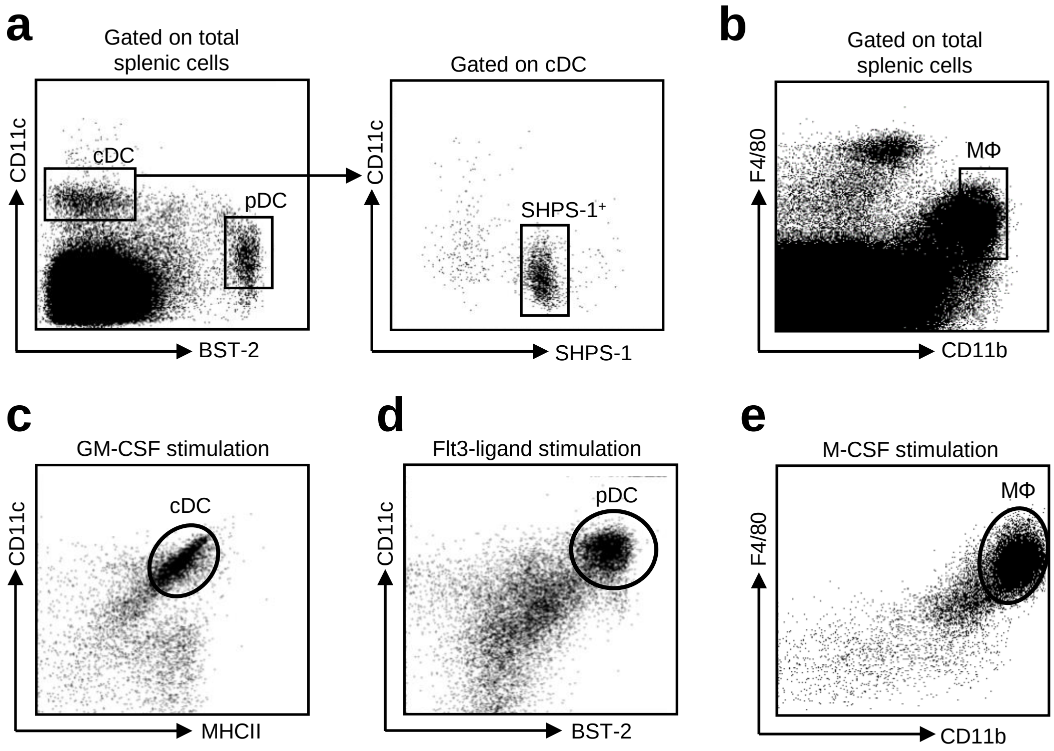

Supplementary Figure 14. Gating strategy used to sort splenic cDCs, pDCs and macrophages from *Ccr2*$^{RFP/+}$ mice and BM-derived cDCs, pDCs and macrophages.

(a,b) Flow cytometry profiles of cDCs (CD11c^{hi}BST-2^{lo}), pDCs (CD11c^{int}BST-2^{hi}) **(a)** and macrophages (F4/80^{+}CD11b^{+}) (**b**) from the spleen of *Ccr2*$^{RFP/+}$ mice. This gating strategy was used to sort DC subsets and macrophages in Fig. 8a,b. **(c-e)** Flow cytometry profiles of BM-derived cDCs (CD11c^{+}MHCII^{+}) **(c)**, pDCs (CD11c^{+}BST-2^{hi}) **(d)** and macrophages (F4/80^{+}CD11b^{+}) **(e)** stimulated with GM-CSF, Flt3-ligand and M-CSF, respectively. This gating strategy was used to sort BM-derived DCs and macrophages in Fig. 8c-e. MΦ; Macrophage.

Supplementary Figure 15

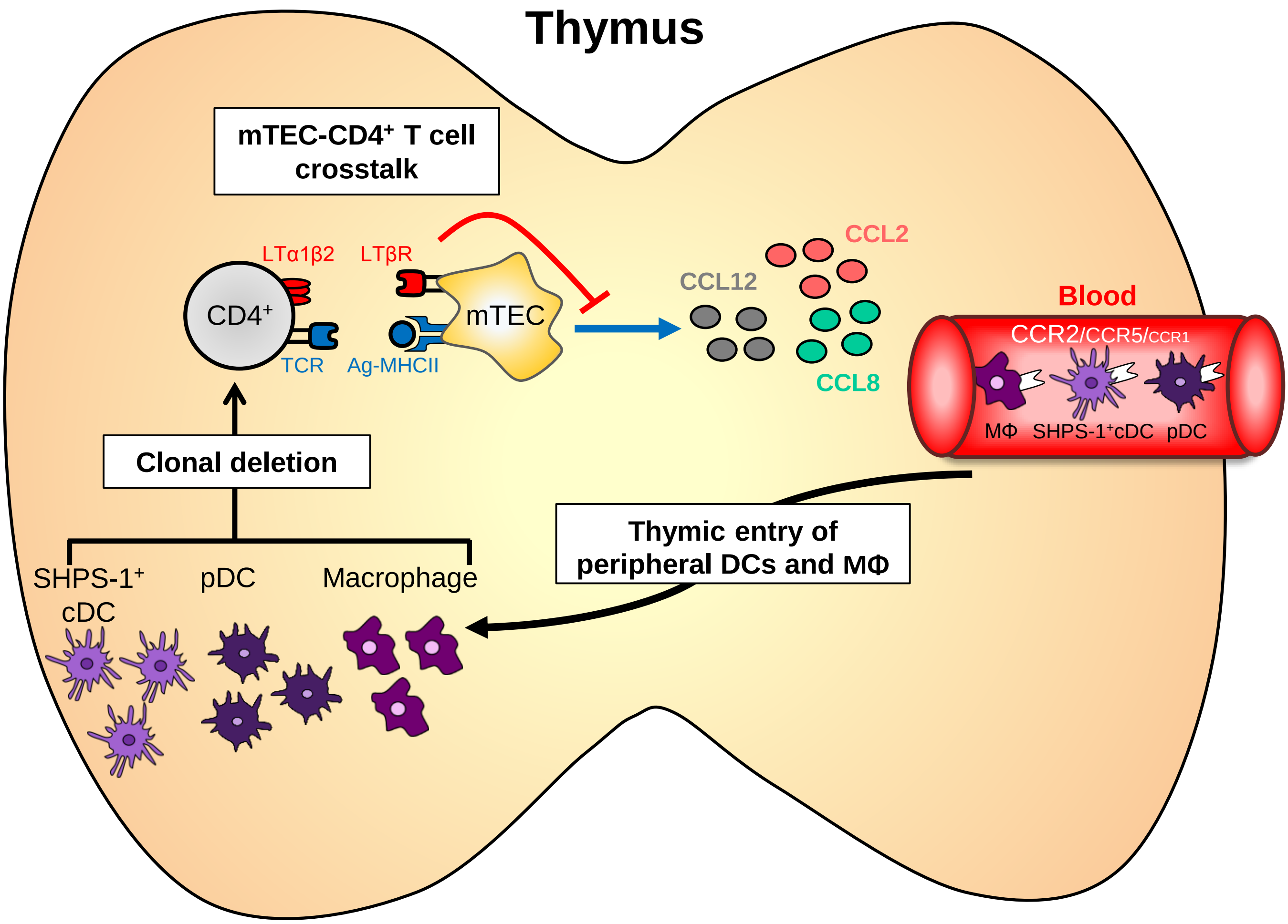

Supplementary Figure 15. LTα induced in CD4⁺ thymocytes by crosstalk with mTECs controls the thymic entry of peripheral DCs and macrophages by negatively regulating CCL2, CCL8 and CCL12 in mTECs, which fine-tunes clonal deletion.

Ag-specific interactions with mTECs induce the upregulation of LTα1β2 in $CD4^+$ thymocytes, which in turn controls the expression of CCL2, CCL8 and CCL12 in $CD80^{lo}$ mTECs. This regulation loop controls, mainly in a CCR2-dependent manner, the recruitment of peripheral DCs and macrophages into the thymus, which tightly regulates clonal deletion.

Supplementary Table 1. Analysis of NF-κB binding sites with MatInspector in the mouse *Ccl12* gene promoter

Matrix Family	Family information	Matrix	Matrix information	Position	Strain	Matrix sim.	Sequence
V$NFKB	Nuclear factor kappa B/c-rel	V$CREL.01	c-Rel	617-631	(-)	0.917	caa**gg**tca**TTCC**tca
V$NFKB	Nuclear factor kappa B/c-rel	V$CREL.01	c-Rel	1065-1079	(+)	0.917	gat**gg**aac**TTCC**agg
V$NFKB	Nuclear factor kappa B/c-rel	V$NFKAPPAB65	NF-kappaB (p65)	1065-1079	(-)	0.916	cct**gg**aa**gTTCC**atc

Supplementary Table 2.
List of antibodies and reagents used

Antibodies	Dilution	Clone	Distributor	Catalogue number
anti-CD11c	1/200	N418	BioLegend	117328
anti-SHPS-1 (anti-Sirpα)	1/200	P84	BioLegend	144024
anti-BST-2 (anti-PDCA-1)	1/200	927	BioLegend	127016
anti-CD8α	1/600	53-6.7	BioLegend	100722
anti-I-Ab (anti-MHCII)	1/600	AF6-1201	BioLegend	116408
anti-CD4	1/200	RM4-5	BioLegend	100529
anti-F4/80	1/200	BM8	BioLegend	123120
anti-TCR Vα2	1/200	B20.1	BioLegend	127808
anti-Helios	1/200	22F6	BioLegend	137222
anti-CCR7	1/100	4B12	BioLegend	120108
anti-CD69	1/400	H1.2F3	BioLegend	104508
anti-PD-1	1/200	29F.1A12	BioLegend	135230
anti-CD45.2	1/200	104	BioLegend	109828
anti-CCR1	1/50	S15040E	BioLegend	152504
anti-CCR5	1/50	HM-CCR5	BioLegend	107006
anti-K14	1/1000	AF64	Covance	PRB-155P-100
anti-NF-κB p65	1/3000	D14E12	Cell Signaling	8242S
anti-phospho NF-κB p65 (Ser536)	1/3000	93H1	Cell Signaling	3033S
anti-RelB	1/200	C-19	SantaCruz Biotechnology	sc-226
anti-MCP1-4	1/100	B-2	SantaCruz Biotechnology	sc-377082
anti-CD62L	1/300	MEL-14	BD Biosciences	553151
anti-CD80	1/200	16-10A1	BD Biosciences	562611
anti-BP-1 (anti-Ly51)	1/3000	BP-1	BD Biosciences	553735
anti-CD45.1	1/800	A20	BD Biosciences	563983
anti-CD11b	1/200	M1/70	BD Biosciences	553312
anti-CD45	1/300	30-F11	BD Biosciences	553081
anti-TCR Vβ5.1, 5.2	1/200	MR9-4	BD Biosciences	553190
anti-CD3	1/200	145-2C11	BD Biosciences	553060
anti-CD19	1/200	1D3	BD Biosciences	553784
anti-CCR2	1/100	475301	RnD systems	FAB5538P-100
anti-Ep-CAM	1/3000	G8.8	ebioscience	25-5791-80
anti-Foxp3	1/150	FJK-16s	ebioscience	12-5773-80
anti-Ki-67	1/200	SolA15	ebioscience	48-5698-82
anti-LTβR	1/200	3C8	ebioscience	13-5671-82
anti-CD16/CD32 (Fc-block)	1 µg/million ce	2.4G2	BD Biosciences	553142
Alexa Fluor 488 Goat anti-Mouse IgG	1/500		Invitrogen	A-11001
Alexa Fluor 647 Donkey anti-Rabbit IgG	1/500		Invitrogen	A-31573
Alexa Fluor 488 Chicken anti-Rabbit IgG	1/500		Invitrogen	A-21441
Cyanine 5 Goat anti-Rabbit IgG	1/500		Invitrogen	A-10523
Alexa Fluor 488 Streptavidin	1/400		Invitrogen	S11223
PerCP Streptavidin	1/200		BioLegend	405213
UEA-1	1/600		Vector Laboratories	FL-1061

Reagents	Distributor	Catalogue number
HBSS (1X)	Thermo Fisher	14175-053
DPBS 10X	Thermo Fisher	14200-067
RPMI Medium 1640	Thermo Fisher	21875-034
Sodium pyruvate	Thermo Fisher	11360-039
2-mercaptoethanol	Thermo Fisher	31350-010
L-glutamine 200mM (10X)	Thermo Fisher	25030-024
penicillin/streptomycin	Thermo Fisher	15140122
FBS	Sigma-Aldrich	F2442
Collagenase D	Sigma-Aldrich	11088866001
DNase I	Sigma-Aldrich	10104159001
1X RBC Lysis buffer	Invitrogen	00-4333-57
CD45 MicroBeads	Miltenyi Biotec	130-052-301
anti-biotin MicroBeads	Miltenyi Biotec	130-090-485
Permeabilization buffer 10X (Foxp3 staining kit)	eBioscience	00-8333-56
Fixation/Permeabilization diluent (Foxp3 staining kit)	eBioscience	00-5223-56
Fixation/Permeabilization concentrate (Foxp3 staining kit)	eBioscience	00-5123-43
Cytoperm/Cytofix	BD Biosciences	51-2090KZ
Perm/Wash Buffer	BD Biosciences	51-2091KZ
Brefeldin A	BioLegend	420601
$OVA_{(323\text{-}339)}$ peptide	Polypeptide group	sc1303
Recombinant murine M-CSF	PeproTech	315-02
Recombinant murine Flt3-ligand	PeproTech	250-31L
Recombinant murine GM-CSF	PeproTech	315-03
O.C.T.	VWR	4583
Mowiol 4-88 Reagent	Calbiochem	475904
DAPI	BioLegend	422801
Paraformaldehyd	Merck	1040051000
TRIzol Reagent	Invitrogen	15596026
Oligo (dT)	life technologies	58862
Superscript II reverse transcriptase	Invitrogen	18064014
SYBR Premix Ex Taq	Takara	RR420L
Recombinant Human LTβR-Fc chimera	RnD systems	7538-LR-100

Supplementary Table 3. List of primers used for qPCR

Gene	Forward (5'-->3')	Reverse (5'-->3')	Publication source
Actin	CAGAAGGAGATTACTGCTCTGGCT	GGAGCCACCGATCCACACA	Edgar AJ, BMC Biochem, 2002, 3:19
Lta	GCTTGGCACCCCTCCTGTC	GATGCCATGGGTCAAGTGCT	Irla M *et al* , Immunity, 2008, 29(3):451-63
Tnfsf11	AGATTTGCAGGACTCGACTC	AGAGTCGAGTCCTGCAAATC	Irla M *et al* , Immunity, 2008, 29(3):451-63
Cd40lg	GTGAGGAGATGAGAAGGCAA	CACTGTAGAACGGATGCTGC	Irla M *et al* , Immunity, 2008, 29(3):451-63
Ccl25	GCCTGGTTGCCTGTTTTGTT	ACCCAGGCAGGCAGCAGTCTTCAA	Rojas-Lopèz AE *et al* , Immunobiology, 2012, 795-807
Ccl2	AGGTCCCTGTCATGCTTCTG	TCATTGGGATCATCTTGCTG	Dvoriantchikova G *et al* , Mol Vis, 2010, 16: 2882-2890
Ccl7	CCTGGGAAGCTGTTATCTTCAA	TGGAGTTGGGGTTTTCATGTC	Nakao J *et al* , Bone, 2014, 58:17-25
Ccl8	GCTGTGGTTTTCCAGACCAA	GAAGGTTCAAGGCTGCAGAA	Nakao J *et al* , Bone, 2014, 58:17-25
Ccl12	ATTTCCACACTTCTATGCCTCCT	ATCCAGTATGGTCCTGAAGATCA	Das A *et al* , Sci Rep, 2015, 5:16932
Relb	GGGCATCCAGTGTGTTAGGAAGAA	GGAAGCAGATCCTGACGACATTCA	Saxon JA *et al* , J immunol, 2016, 196(4):1891-9
Rel	GTGTCAGGGGAGGAGATGAA	CTCTGGCTTCCCAGTCATTC	Home made designed
Rela	TGGCGAGAGAAGCACAGATA	CCTGGTCCTGTGTAGCCATT	Shalini S and Bansal MP, Biometals, 2007, 20(1):49-59
